# The Interplay of Tobacco Farming and Tobacco Control: Exploring Socioeconomic and Health Dynamics in Malawi

**DOI:** 10.1002/puh2.70008

**Published:** 2024-10-30

**Authors:** Chimwemwe Ngoma, Sahan Lungu, George N. Chidimbah Munthali, Martha Shantel Mwase

**Affiliations:** ^1^ Tobacco Harm Reduction Scholarship Programme Knowledge Action Change London UK; ^2^ ThinkSmart Consulting Lilongwe Malawi; ^3^ Independent Researcher; ^4^ Faculty of Environmental Sciences Mzuzu University Mzuzu Malawi

**Keywords:** health implications, Malawi, socioeconomic dynamics, tobacco control, tobacco endgame, tobacco farming

## Abstract

Tobacco farming and tobacco control are closely inter‐related issues, particularly in developing countries that are heavily reliant on tobacco farming like Malawi. This commentary explores the relationship among tobacco farming, economic sustainability, and public health, focusing on the context of Malawi as one of the major tobacco producers in Africa. Malawi's economy is significantly dependent on tobacco farming, yet the country also struggles with smoking‐related health risks, creating a dilemma between socioeconomic and public health considerations. The commentary highlights the lack of empirical evidence regarding the socioeconomic implications of tobacco control measures on tobacco farming in the country. Moreover, despite the country's economic dependence on tobacco farming, a majority of cigarettes consumed domestically are imported, demonstrating the complexity of Malawi's tobacco industry. On public health implications, the article highlights the disease and death burden that is a result of tobacco smoking, underscoring the need for tobacco control measures. The article further draws insights from tobacco end‐game strategies in other countries and proposes a comprehensive approach to tobacco control. However, the article notes Malawi's limited financial resources and healthcare infrastructure to implement traditional tobacco control measures and highlights an emphasis on tobacco harm reduction, a third pillar of the Framework Convention on Tobacco Control. The article also advocates for an emphasis on alternative livelihood opportunities for smallholder tobacco farmers in Malawi.

## 1 Background

Tobacco farming and tobacco control have emerged as closely intertwined subjects of contemporary interest. Countries in the developing world that depend mostly on agriculture, especially on tobacco farming as their primary sources of income have been much affected by this interplay. For instance, Malawi, one of the largest tobacco producers in Africa [1], faces unique challenges in addressing the harmful effects of tobacco use while relying on tobacco farming for economic sustainability.

Despite the interconnectedness, there is an apparent lack of collaboration among government ministries involved in tobacco‐related issues. The Ministry of Agriculture oversees farming, the Ministry of Trade manages exports, and health issues are handled by the Ministry of Health. This fragmented approach is not unique to Malawi, and tobacco control policymaking often encounters opposition between health ministry and finance, agriculture, and trade ministries in many Sub‐Saharan African countries [2].

Although acknowledging the importance of addressing the health effects of tobacco use, it is worth noting that until August 2023, Malawi was one of the few countries that were not a party to the World Health Organization's (WHO) Framework Convention on Tobacco Control (FCTC). Additionally, tobacco control measures, including smoking cessation, have been opposed by some stakeholders in the country. A frequent justification for the opposition is the preservation of the economic viability of tobacco farmers [3]. This argument suggests that promoting either tobacco farming or tobacco control in the country could be in conflict, as these two goals are thought to be at opposite ends of the spectrum.

However, there is a lack of evidence to support the claim that promoting tobacco control measures in countries reliant on tobacco growing for income generation has significant socioeconomic implications on tobacco farming. Intriguingly, a majority of cigarettes consumed domestically in Malawi are imported; for instance, 421 million cigarettes were imported versus 42 million produced domestically in 2016 [4]. In contrast, neighboring Zambia produces approximately 25 million cigarettes daily, with 20 to 22 million intended for the export market [5]. Meanwhile, Malawi predominantly generates its revenue by exporting raw tobacco to the global market.

This commentary highlights the importance of a balanced understanding of the interplay between tobacco farming and tobacco control to contribute to sustainable development where economic growth and public health are harmoniously intertwined.

### 1.1 Economic Role of Tobacco Farming

According to the World Bank's 2022 Poverty Assessment for Malawi, agriculture remains the cornerstone of the country's economy, sustaining approximately 85% of its nearly 20 million people [6]. Malawi's economy as a whole and farmers’ livelihoods are significantly impacted by tobacco growing [7]. Notably, in the year 2017, which serves as the most recent point of comprehensive analysis, the exportation of raw tobacco constituted a substantial 52% of Malawi's export earnings, contributing a noteworthy 10.2% to the country's gross domestic product (GDP) [7]. These figures underscore the crucial economic role of tobacco leaf production in Malawi. Additionally, tobacco cultivation has historically supported Malawian families in educating their children, building homes, providing employment, and earning a living, thereby amplifying its socioeconomic impact, particularly in rural settings where other economic opportunities are limited [8].

### 1.2 Cultural and Agrarian Significance

The agricultural landscape in Malawi, especially for certain farmers, is intertwined with tobacco cultivation. For many individuals engaged in tobacco farming, the crop represents not only a significant source of income but also an intrinsic facet of their agricultural expertise, cultivated over a lifetime [9]. The persistence of tobacco cultivation can be attributed to several interrelated factors. First, tobacco's reputation as a high‐yielding crop encourages its continued cultivation and provides farmers with financial stability [10]. For many, it is their only source of cash income. Second, tobacco's resilience to adverse weather conditions increases its attractiveness and provides a degree of agricultural security [10]. Third, the well‐established tobacco market infrastructure creates further incentives for tobacco cultivation, thus ensuring a reliable sales channel. Finally, the creditworthiness associated with tobacco production strengthens cultivation as farmers have easier access to finance and resources [10], enhancing economic viability. These factors collectively contribute to the continued presence of tobacco farming within the agricultural fabric of Malawi, simultaneously impeding the adoption of alternative crops or economic pursuits.

However, tobacco production in Malawi has witnessed a significant decline, falling from 16% to 5% of Malawian crop farmers between 2004 and 2019 [11]. This could be attributed, in part, to the global decline in tobacco consumption, along with a decreased demand on the global market [12].

### 1.3 Public Health Implications

Despite the economic and cultural significance of tobacco farming, tobacco use has public health implications. In many countries, tobacco smoking is one of the main contributors to the burden of disease [13]. Annually, smoking claims 8 million lives and affects people with chronic non‐communicable diseases such as heart disease, lung disease, cancer, diabetes, and high blood pressure [14]. In the year 2019, Malawi witnessed 5403 deaths directly attributed to smoking [15]. Additionally, smoking contributed to the accumulation of 174,616 disability‐adjusted life years [15]. This development underscores the need to address tobacco smoking as a priority public health problem, requiring robust interventions that protect the well‐being of communities.

Additionally, the 2017 STEPwise approach to surveillance (STEPS) survey illuminates the prevalence of tobacco use in Malawi to be at 9%, revealing a notably higher occurrence among males (21%) in contrast to females (2%) [16]. Similar patterns are observed in other Southern African countries that also produce tobacco. In Zambia, the overall smoking prevalence is 11%, with male prevalence at 25.7% and female prevalence at 2.2% [17]. Zimbabwe presents a similar picture, with an overall prevalence of 11.8%, a male prevalence of 21.8%, and a female prevalence of 1.5% [18, 19]. These figures demonstrate the widespread nature of tobacco use in these countries and the gender disparities in smoking prevalence.

Paradoxically, despite the noticeable health consequences of smoking in Malawi, little attention has been paid to effectively address the situation. This inadequacy partly stems from the limited funds allocated to tobacco control efforts, a dilemma exacerbated by the underfunded state of the healthcare system [20]. The lack of funding has presented challenges to the implementation of comprehensive and sustained tobacco control projects, thereby perpetuating an unfortunate gap in efforts to reduce tobacco‐related health risks. This discrepancy underscores the urgent need for a concerted effort to increase financial support for tobacco control initiatives while strengthening health infrastructure to pave the way for effective and low‐cost interventions that can effectively alleviate the burden of smoking‐related diseases in the Malawian population.

### 1.4 Recommendations for Addressing Tobacco Control and Economic Development

In light of Malawi's juxtaposition between tobacco farming and the health effects of smoking, achieving a balance between socioeconomic and health dynamics requires the formulation of innovative and comprehensive strategies that involve a careful reassessment of the prevailing economic dependencies. This article proposes the following approaches.

#### 1.4.1 Promoting Alternatives to Tobacco Production

The global demand for tobacco is declining due to increased awareness of its health risks [12]. In the long run, this trend threatens the economic stability of countries heavily reliant on tobacco exports. To mitigate this risk, it is important to scale up the existing initiatives aimed at diversifying agricultural practices and livelihood opportunities [7], particularly those tailored to empower smallholder tobacco farmers. This strategy is consistent with efforts in other tobacco‐dependent countries like India where high‐value crop alternatives are being promoted [21].

#### 1.4.2 Learning From Global Tobacco Control Strategies

Implementing tobacco control strategies plays an important role in tackling the health effects of smoking. Malawi can draw lessons from the tobacco endgame strategies in other countries. For instance, Australia's plain packaging laws have been successful in reducing the appeal of tobacco products and discouraging smoking initiation. Additionally, increasing tobacco taxes, as implemented by France and the United Kingdom, has proven to be an effective method of reducing tobacco consumption. Furthermore, comprehensive smoke‐free policies in public spaces and workplaces, as seen in Ireland, can help reduce exposure to secondhand smoke [22, 23, 24].

Unfortunately, implementing tobacco control strategies is expensive and is hindered by the governments’ inadequate financial support [3, 20]. This necessitates a benefit–‐cost analysis to determine whether the potential benefits of tobacco control efforts justify the costs, and how these efforts compare with other government expenditure options. Understanding the return on investment for tobacco control compared to other public health or economic initiatives can inform decisions that optimize resource allocation.

#### 1.4.3 Emphasizing Harm Reduction

For countries operating under resource constraints like Malawi, an increased focus on harm reduction, which constitutes the third pillar of tobacco control as delineated in Article 1d of the FCTC, represents a pragmatic and important strategy among current smokers who are unable or unwilling to give up on smoking. The adoption of tobacco harm reduction products helps in reducing smoking prevalence faster than traditional tobacco control measures solely focused on prevention and cessation [25]. This is evident in countries, like Sweden and Norway, where the availability of snus, a tobacco harm reduction product, has contributed to the unusually low rates of smoking by helping consumers switch to a less harmful form of nicotine dependence [26, 27]. Furthermore, implementing harm reduction strategies incurs minimal costs to the government and taxpayers. Manufacturers shoulder the research and development costs, whereas consumers fund their transition to safer nicotine products, thus moving away from high‐risk tobacco products [28].

#### 1.4.4 Coordinating Policy Development

In pursuit of these strategic goals, promoting shared policy interventions and informed public discourse could be a promising and effective approach. Establishing an inter‐ministerial committee comprising the health, agriculture, finance, and trade ministries to oversee the various aspects of tobacco could ensure well‐coordinated policy development that addresses both economic and public health objectives. Importantly, integrating the perspectives of smallholder tobacco farmers and nicotine consumers, who are among the most directly affected stakeholders, is crucial. By utilizing the synergies between these elements, Malawi can embark on a definitive path toward harmonizing its aspirations for economic prosperity and the fundamental imperative of maintaining public health.

## 2 Conclusion

The intertwined narratives of tobacco cultivation and tobacco control in Malawi reflect the broader global challenge of balancing economic growth with maintaining good health. This commentary serves as a clear call to recognize and address the dynamics underlying this interplay. As Malawi navigates these complex waters, it has an opportunity not only to forge its own path but also to contribute to a broader global discussion on sustainable development, based on both economic prosperity and well‐being.

## Author Contributions

The original concept for this commentary was conceived by Chimwemwe Ngoma and took the lead in composing the initial draft. Subsequently, George N. Chidimbah Munthali, Chimwemwe Ngoma, Martha Shantel Mwase, and Sahan Lungu engaged in a critical revision process to enhance its intellectual depth. All authors participated in reviewing, reading, and endorsing the final version of the article.

## Ethics Statement

The authors have nothing to report.

## Conflicts of Interest

Chimwemwe Ngoma is a consultant to Knowledge•Action•Change (K•A•C). K•A•C is a public health organization with a focus on tobacco harm reduction and is funded with a grant from Global Action to End Smoking (formerly known as Foundation for Smoke‐Free World), an independent, US nonprofit 501(c)(3) grantmaking organization, accelerating science‐based efforts worldwide to end the smoking epidemic. Sahan Lungu is a Kevin Molloy Fellow and Martha Shantel Mwase is a scholar in the Tobacco Harm Reduction Scholarship Programme (THRSP) by K•A•C. The views and opinions expressed in the contents of this article are the sole responsibility of the authors, and should not, under any circumstances, be regarded as reflecting the positions of K•A•C. George N. Chidimbah Munthali declares no competing interests.

## Data Availability

All sources used have been properly cited.
